# Quantitative Determination of Cholesterol Hydroxylase Specificities by GC–MS/MS in Living Mammalian Cells

**DOI:** 10.21769/BioProtoc.4924

**Published:** 2024-01-20

**Authors:** Hodaka Saito, Mizuki Nishimura, Ryuichiro Sato, Yoshio Yamauchi

**Affiliations:** 1 Laboratory of Food Biochemistry, Department of Applied Biological Chemistry, Graduate School of Agricultural and Life Sciences, The University of Tokyo, Tokyo, Japan; 2 Nutri-Life Science Laboratory, Department of Applied Biological Chemistry, Graduate School of Agricultural and Life Sciences, The University of Tokyo, Tokyo, Japan; 3 AMED-CREST, Japan Agency for Medical Research and Development, Tokyo, Japan

**Keywords:** Cholesterol, Oxysterols, Intermediate sterols, Cholesterol hydroxylase, GC–MS/MS, CH25H, CYP27A1, CYP46A1, CYP7A1

## Abstract

Cholesterol is oxygenated by a variety of cholesterol hydroxylases; oxysterols play diverse important roles in physiological and pathophysiological conditions by regulating several transcription factors and cell-surface receptors. Each oxysterol has distinct and overlapping functions. The expression of cholesterol hydroxylases is highly regulated, but their physiological and pathophysiological roles are not fully understood. Although the activity of cholesterol hydroxylases has been characterized biochemically using radiolabeled cholesterol as the substrate, their specificities remain to be comprehensively determined quantitatively. To better understand their roles, a highly sensitive method to measure the amount of various oxysterols synthesized by cholesterol hydroxylases in living mammalian cells is required. Our method described here, with gas chromatography coupled with tandem mass spectrometry (GC–MS/MS), can quantitatively determine a series of oxysterols endogenously synthesized by forced expression of one of the four major cholesterol hydroxylases—CH25H, CYP7A1, CYP27A1, and CYP46A1—or induction of CH25H expression by a physiological stimulus. This protocol can also simultaneously measure the amount of intermediate sterols, which serve as markers for cellular cholesterol synthesis activity.

Key features

• Allows measuring the amount of a variety of oxysterols synthesized endogenously by cholesterol hydroxylases using GC–MS/MS.

• Comprehensive and quantitative analysis of cholesterol hydroxylase specificities in living mammalian cells.

• Simultaneous quantification of intermediate sterols to assess cholesterol synthesis activity.

**Graphical overview**


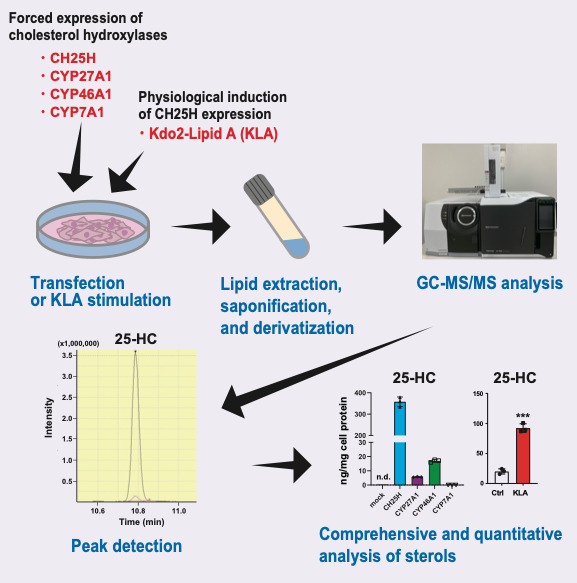


## Background

Cholesterol plays diverse important biological roles, including function regulation of biological membranes and membrane proteins, and is the precursor for steroid hormones and bile acids; thus, cellular cholesterol homeostasis is tightly controlled by multiple mechanisms (Chang et al., 2006; Yamauchi and Rogers, 2018; Luo et al., 2020). Mammalian cells cannot break down the sterol backbone. Instead, cholesterol is converted to cholesteryl ester for storage (Chang et al., 2006) and to various oxysterols (Mutemberezi et al., 2016). Oxysterols exert different functions depending on the hydroxylation site(s). Multiple oxysterols whose side chain is hydroxylated, including 25-hydroxycholesterol (25-HC) and 27-HC, regulate cellular cholesterol homeostasis; they modulate two important transcription factors: sterol regulatory element-binding protein-2 (SREBP-2) and liver X receptor (LXR) (Gill et al., 2008; M.S. Brown et al., 2018). SREBP-2 transactivates most genes involved in cholesterol acquisition (biosynthesis and uptake) (Horton et al., 2002). On the other hand, LXR upregulates the expression of several ATP-binding cassette (ABC) transporters including ABCA1 and ABCG1 that mediate the export of excess cellular cholesterol (Tontonoz, 2011). Mechanistically, side-chain oxysterols bind to Insig-1 and Insig-2—retention factors for SREBP-2 in the endoplasmic reticulum (ER)—and protect them from proteasomal degradation, thereby inhibiting SREBP-2 activation. Side-chain oxysterols also serve as natural LXR ligands when added exogenously.

**Figure 1.**
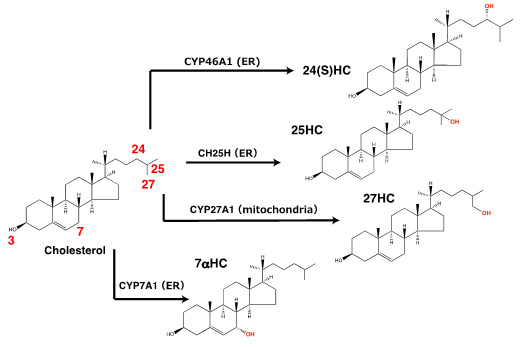
Hydroxylation of cholesterol by the four cholesterol hydroxylases handled in this protocol. CH25H, CYP27A1, CYP46A1, and CYP7A1 mainly produce 25-HC, 27-HC, 24(S)-HC, and 7α-HC, respectively.

A series of hydroxylases catalyze the site-specific hydroxylation of cholesterol (Mutemberezi et al., 2016). Although a variety of oxysterols have been identified in the body, 7α-HC, 27-HC, 24S-HC, and 25-HC are the most abundant. These four oxysterols are synthesized largely by CYP7A1, CYP27A1, CYP46A1, and CH25H, respectively, in the ER or mitochondria (Figure 1), while some of these hydroxylases also produce other oxysterols as minor products (Mutemberezi et al., 2016; Saito et al., 2023). Recent studies show that the expression of cholesterol hydroxylases is highly regulated in physiological and pathophysiological conditions (Cyster et al., 2014; A.J. Brown et al., 2021). CH25H expression and 25-HC biosynthesis are markedly upregulated upon infection in immune cells such as macrophages, and 25-HC itself exhibits anti-bacterial and anti-viral effects, protecting cells from infection (Bauman et al., 2009; S.Y. Liu et al., 2013; Zhou et al., 2020). 25-HC can be further hydroxylated at the 7-position by the hydroxylase CYP7B1, generating 7α,25-dihydroxycholesterol (7α,25-diHC). This dihydroxycholesterol is a ligand for the G-protein coupled receptor GPR183 (also known as EBI2) involved in immune cell migration (C. Liu et al., 2011). Higher circulating 27-HC levels associate with the risk of estrogen receptor-positive breast cancer, since 27-HC serves as an endogenous selective estrogen receptor modulator (Nelson et al., 2013). CYP46A1 is a neuron-specific cholesterol hydroxylase that converts cholesterol into 24S-HC for eliminating excess cholesterol in the brain (Russell et al., 2009). CYP7A1 is a liver-specific hydroxylase that serves as the rate-limiting enzyme for bile acid synthesis (Russell, 2009). Although the activity of these cholesterol hydroxylases is biochemically studied using radiolabeled cholesterol as the substrate, comprehensive and quantitative characterization of their specificities remains poorly explored in living cells. Furthermore, precise measurement of the products of these cholesterol hydroxylases is crucial for better understanding their physiological and pathophysiological roles.

Here, we provide a highly sensitive method using gas chromatography coupled with tandem mass spectrometry (GC–MS/MS) to determine various oxysterols synthesized in living mammalian cells where a cholesterol hydroxylase is forcedly expressed or is upregulated by lipopolysaccharide, a component of Gram-negative bacteria known to upregulate CH25H expression in macrophages (Bauman et al., 2009). The current GC–MS/MS protocol enabled us to reveal cholesterol hydroxylase–specific production of oxysterols (Saito et al., 2023). Our protocol can also simultaneously determine the amount of intermediate sterols to monitor the activity of cholesterol biosynthesis. Accordingly, our GC–MS/MS-based sterol analysis, in combination with biochemical and gene expression studies, has suggested that side-chain oxysterols enzymatically synthesized within cells primarily regulate SREBP-2 but not LXR (Saito et al., 2023).

## Materials and reagents

1. 1.5 mL sampling tubes (WATSON, catalog number: 131-8155C)
2. Screw-top test tube (Maruemu, catalog number: NR-10)
3. Mighty Vials (Maruemu, catalog number: 84-0561)
4. TORAST vial (Shimadzu GLC, catalog number: GLCTV-902)
5. TORAST vial insert (Shimadzu GLC, catalog number: GLCTV-I04)
6. TORAST vial cap (Shimadzu GLC, catalog number: GLCTV-903)
7. Hexane (Fuji Film Wako, catalog number: 085-00416)
8. 2-propanol (Fuji Film Wako, catalog number: 166-04836)
9. Dibutyl hydroxytoluene (BHT) (Fuji Film Wako, catalog number: 029-07392)
10. Nitrogen gas
11. Helium gas
12. Argon gas
13. Chloroform (Fuji Film Wako, catalog number: 038-02606)
14. Ethanol (Fuji Film Wako, catalog number: 057-00456)
15. Milli-Q water
16. Pyridine (Fuji Film Wako, catalog number: 161-18453)
*Note: Store pyridine in a desiccator with silica gel at room temperature and use within four weeks after opening.*
17. N-methyl-N-trimethylsilyl trifluoroacetamide (MSTFA) (GL Science, catalog number: 1022-11061)
18. Cholesterol (purity ≥ 99%) (Sigma-Aldrich, catalog number: C8667)
19. Cholesterol-d7 (purity ≥ 99%) (Avanti Polar Lipids, catalog number: 700041P)
20. Desmosterol (purity ≥ 99%) (Nagara Science, catalog number: NS460402)
21. Lanosterol (purity ≥ 99.5%) (Nagara Science, catalog number: NS460102)
22. Lathosterol (purity ≥ 99%) (Nagara Science, catalog number: NS460502)
23. 7-dehydrocholesterol (purity ≥ 95%) (Sigma-Aldrich, catalog number: 30800)
24. 24,25-epoxycholesterol (purity ≥ 95%) (Abcam, catalog number: Ab141633)
25. 24,25-dihydrolanosterol (purity ≥ 99.5%) (Nagara Science, catalog number: NS460201)
26. 4β-hydroxycholesterol (purity ≥ 95%) (Cayman, catalog number: 19518)
27. 7α-hydroxycholesterol (purity ≥ 99%) (Avanti Polar Lipids, catalog number: 700034P)
28. 7β-hydroxycholesterol (purity ≥ 95%) (Sigma-Aldrich, catalog number: H6891)
29. 7α,25-dihydroxycholesterol (purity ≥ 98%) (Sigma-Aldrich, catalog number: SML0541)
30. 7α,27-dihydroxycholesterol (purity ≥ 99%) (Avanti Polar Lipids, catalog number: 700024P)
31. 25-hydroxycholesterol (purity ≥ 98%) (Sigma-Aldrich, catalog number: H1015)
32. 24(S)-hydroxycholesterol (purity ≥ 98%) (Sigma-Aldrich, catalog number: SML1648)
33. 25-hydroxycholesterol-d6 (Avanti Polar Lipids, catalog number: LM4113-1EA)
34. 27-hydroxycholesterol (purity ≥ 98%) (Sigma-Aldrich, catalog number: SML2042)
35. Sodium chloride (Fuji Film Wako, catalog number: 196-01665)
36. Potassium chloride (SIGMA, catalog number: P9541)
37. Disodium hydrogen phosphate dodecahydrate (Fuji Film Wako, catalog number: 196-02835)
38. Potassium dihydrogen phosphate (Fuji Film Wako, catalog number: 196-04245)
39. Potassium hydroxide (Fuji Film Wako, catalog number: 168-21815)
40. Sodium hydroxide (Fuji Film Wako, catalog number: 192-15985)
41. D-MEM/Ham’s F-12 with L-Glutamine and Phenol Red (Fuji Film Wako, catalog number: 048-29785)
42. RPMI-1640 with L-Glutamine and Phenol Red (Fuji Film Wako, catalog number: 189-02025)
43. Opti-MEM^TM^ I reduced serum medium (Thermo Fisher Scientific, catalog number: 31985070)
44. Fetal bovine serum (FBS) (lot number: 27419002) (Corning, catalog number: 35-079-CF)
45. Penicillin G potassium (Meiji Seika Pharma, catalog number: 6111400D3051)
46. Streptomycin sulfate (Meiji Seika Pharma, catalog number: 6161400D1034)
47. Lipofectamine LTX reagent (Thermo Fisher Scientific, catalog number: 15338100)
48. Doxycycline hyclate (LKT Labs, catalog number: D5897)
49. Kdo2-Lipid A (Avanti Polar Lipids, catalog number: 69500P)
50. Pierce BCA Protein Assay kit (Thermo Fisher Scientific, catalog number: 23227)

**Biological materials**

1. CHO-K1 cells (gift from Dr. Ta-Yuan Chang, Geisel School of Medicine at Dartmouth)
2. CHO-CH25H^tet-on^ cells (Saito et al., 2023)
3. J774.1 cells (RIKEN Cell Bank, catalog number: RCB0434)
4. Expression plasmids encoding cholesterol hydroxylase (Saito et al., 2023): pFLAG-CH25H, pCYP27A1-FLAG, pCYP46A1-FLAG, and pCYP7A1-FLAG
*Note: Detailed information on CHO-CH25H^tet-on^ cells and the four hydroxylase expression plasmids are described in the original paper (Saito et al., 2023).*

**Solutions**

1. 70% Ethanol (see Recipes)
2. 75% Ethanol (see Recipes)
3. 10 N KOH (dissolved in 75% ethanol) (see Recipes)
4. Hexane/2-propanol (3:2) with 0.01% BHT (see Recipes)
5. 0.1 N NaOH (see Recipes)
6. PBS (see Recipes)

**Recipes**

1. **70% Ethanol**
  **Table:** 

Reagent	Volume
Ethanol (absolute)	35 mL
H_2_O	15 mL
Total	50 mL
2. **75% Ethanol**
  **Table:** 

Reagent	Volume
Ethanol (absolute)	37.5 mL
H_2_O	12.5 mL
Total	50 mL
3. **10 N KOH (dissolved in 75% ethanol)**
  **Table:** 

Reagent	Quantity
Potassium hydroxide	28.1 g
75% Ethanol	up to 50 mL
Total	50 mL
4. **Hexane/2-propanol (3:2) with 0.01% BHT**
  **Table:** 

Reagent	Quantity
Hexane	60 mL
2-propanol	40 mL
Dibutyl hydroxytoluene	10 mg
Total	100 mL
5. **0.1 N NaOH**
  **Table:** 

Reagent	Quantity
Sodium hydroxide	0.2 g
H_2_O	up to 50 mL
Total	50 mL
6. **PBS**
  **Table:** 

Reagent	Quantity
Sodium chloride	8 g
Potassium chloride	0.2 g
Disodium hydrogen phosphate dodecahydrate	3.6 g
Potassium dihydrogen phosphate	g
H_2_O	up to 1 L
Sodium hydroxide	1 L

## Equipment

1. GC–MS/MS (Shimadzu, model: GCMS-TQ8040NX) equipped with an auto-sampler (AOC-20i)
2. BPX5 GC capillary column (30 m × 0.25 mm, 0.25 μm) (TRAJAN, catalog number: SGE-054101)
3. Rxi Guard column (5 m × 0.25 mm) (Restek, catalog number: 10029)
4. Eppendorf ThermoMixer C (Eppendorf, catalog number: 5382000023)
5. Hamilton micro syringe 710RN (Hamilton, catalog number: 72-5004)
6. Pressured gas blowing concentrator (EYELA, model: MGS-2200)
7. Aluminum block MGB-1540 (EYELA, catalog number: 207580)
8. Aluminum block MGB-1624 (EYELA, catalog number: 207610)
9. Low-speed refrigerated centrifuge (TOMY, model: AX-511)
10. SpectraMax (Molecular Devices, model: M2e)

## Software and datasets

1. GC-MSsolution v4 (Shimadzu)
2. RStudio software (Posit)
3. GraphPad Prism 9 (GraphPad)

## Procedure

- **Cell culture**
  **Preparation of CHO-K1 cells expressing cholesterol hydroxylase**
  1. Seed CHO-K1 cells in triplicate into 6-well plates at a density of 2 × 10^5^ cells per well and incubate in DMEM/Ham’s F-12 medium supplemented with 7.5% FBS, penicillin (100 unit/ mL), and streptomycin (0.1 mg/mL) at 37 °C and 5% CO_ 2_ for 18–24 h.
  2. Change the medium to Opti-MEM 2–3 h before transfection.
  3. Transfect cells with 2 μg of plasmid encoding either CH25H, CYP7A1, CYP27A1, or CYP46A1 (pFLAG-CH25H, pCYP7A1-FLAG, pCYP27A1-FLAG, or pCYP46A1-FLAG, respectively) using Lipofectamine LTX reagent according to the manufacturer’s protocol.
*Note: These four hydroxylase expression constructs are described in the original paper (Saito et al., 2023).*
  4. Change the medium to DMEM/Ham’s F-12 medium containing 0.1% FBS, penicillin (100 unit/ mL), and streptomycin (0.1 mg/mL) 5 h after transfection.
  5. Incubate cells for 24 h at 37 and 5% CO_2_ before harvesting cells for lipid extraction.
  **Preparation of CHO-CH25H^tet-on^ cells**
  1. Plate 2 × 10^5^ CHO-CH25H^tet-on^ cells in triplicate into a well of a 6-well plate and incubate for 18–24 h in DMEM/Ham’s F-12 medium with 7.5% FBS, penicillin (100 unit/ mL), and streptomycin (0.1 mg/mL) at 37 °C and 5% CO_2_.
  2. Treat the cells for 24 h with or without 0.4 or 1 μg/mL of doxycycline hyclate in DMEM/Ham’s F-12 medium containing 0.1% FBS and penicillin/streptomycin to induce FLAG-CH25H expression and harvest the cells to extract cell lipids.
  **Preparation of J774.1 cells**
  1. Seed J774.1 cells in triplicate into 6-well plates at a density of 5 × 10^5^ cells per well and incubate for two days in RPMI-1640 medium supplemented with 10% FBS and penicillin/streptomycin at 37 and 5% CO_2_.
  2. Incubate the cells with or without 100 ng/mL of Kdo2-Lipid A, a toll-like receptor 4 ligand, in RPMI-1640 medium containing 10% FBS and penicillin/streptomycin for 20 h at 37 before extracting cell lipids.
- **Extraction of cellular lipids**
  1. Wash cells twice in 6-well plates with PBS (1.5 mL/well) and let cells dry at room temperature.
  2. Add 1.5 mL/well of hexane/2-propanol (3:2) with 0.01% BHT (see Recipes) into 6-well plates and place at room temperature for 1 h to extract cellular lipids.
  3. Collect hexane/2-propanol into a glass tube (screw-top test tube).
  4. Add 1 mL/well of hexane/2-propanol (3:2) with 0.01% BHT into the same well and place at room temperature for 30 min.
  5. Transfer hexane/2-propanol into the same glass tube to pool the cellular lipids extracted.
  6. Add cholesterol-d7 (100 ng/tube from 5 μg/mL stock in chloroform) and 25-hydroxycholesterol-d6 (10 ng/tube from 1 μg/mL stock in methanol) to each sample as internal controls.
  7. Evaporate the organic solvent under the nitrogen gas stream at room temperature using a pressured gas blowing concentrator MGS-2200.
  8. Add 1 mL of 100% ethanol and 300 μL of 10 N KOH (dissolved in 75% ethanol) (see Recipes) to each tube, close with a screw cup, and mix gently.
  9. Incubate the tubes at 80 °C for 1 h to saponify lipids.
  10. Cool the tubes on ice.
  11. Add 2 mL of chloroform into each tube and vortex.
  12. Add 2.5 mL of distilled water into each tube and vortex.
  13. Centrifuge the tubes at 2,380× *g* for 10 min at room temperature using the low-speed refrigerated centrifuge AX-511.
  14. Remove the aqueous phase (upper layer) using an aspirator.
  15. Add 2 mL of distilled water into each tube again and vortex for 1 min.
  16. Centrifuge the tubes at 2,380× *g* for 10 min at room temperature.
  17. Remove the aqueous layer again using an aspirator.
  18. Add 2 mL of distilled water into each tube again, vortex, and centrifuge at 2,380× *g* for 10 min at room temperature.
  19. Remove the aqueous layer using an aspirator.
  20. Transfer the chloroform phase (which contains non-saponified lipids, including sterols) to a new glass vial (Mighty Vials) using a Pasteur pipette and dry under nitrogen gas stream.
- **Derivatization of extracted cellular sterols**
  1. Add 50 μL of pyridine and 50 μL of MSTFA into each vial and mix gently.
  2. Incubate the vials at 80 °C for 1 h with agitation at 300 rpm using an Eppendorf ThermoMixer C.
  3. Transfer the derivatized sample to a new TORAST vial with a TORAST vial insert using a Hamilton micro syringe and close the tube with a TORAST vial cap.
- **Preparation of standard sterol mixtures**
  1. Prepare two types of standard sterol mixtures in separate vials, as follows: the standard mixtures of non-hydroxysterols contain cholesterol, cholesterol-d7, desmosterol, 7-dehydrocholesterol, lathosterol, lanosterol, and 24,25-dihydrolanosterol; those of oxysterols contain 4β-hydroxycholesterol, 7α-hydroxycholesterol, 7β-hydroxycholesterol, 24(S)-hydroxycholesterol, 25-hydroxycholesterol, 25-hydroxycholesterol-d6, 27-hydroxycholesterol, 7α,25-dihydroxycholesterol, 7α,27-dihydroxycholesterol, and 24,25-epoxycholesterol. The quantities of each sterol added to each vial are 0.1, 1, 5, 10, 50, and 100 ng for the non-hydroxysterol mixtures and 0.01, 0.1, 0.5, 1, 5, 10, and 20 ng for the oxysterol mixtures.
  2. Dry up under nitrogen gas stream at room temperature.
  3. Dissolve sterols in 50 μL of pyridine and 50 μL of MSTFA.
*Note: The final concentrations are 1, 10, 50, 100, 500, and 1000 ng/mL for the non-hydroxysterol mixtures and 0.1, 1, 5, 10, 50, 100, and 200 ng/mL for the oxysterol mixtures.*
  4. Incubate the vials at 80 °C for 1 h with agitation at 300 rpm using an Eppendorf ThermoMixer C.
  5. Transfer the derivatized sample to a new TORAST vial with a TORAST vial insert using a Hamilton micro syringe and close the tube with a TORAST vial cap.
- **GC–MS/MS analysis**
  1. Inject 1 μL of sample or the standard mixture into a GCMS-TQ8040 NX equipped with a BPX5 GC column using an AOC-20i autosampler in splitless mode. Detailed conditions for the analysis are described in Table 1 .
    **Table 1.** GC–MS/MS conditions

GC parameters	
Injection mode	Spitless
Column	BPX5 GC column (30 m × 0.25 mm, 0.25 mm) Rxi Guard Column (5 m × 0.25 mm)
Carrier gas control	Linear velocity (49.5 cm/s)
High pressure injection	250 kPa (1 min)
Injection temperature	275 °C
Column oven temperature	200 °C (1 min) → 25 °C/min → 250 °C → 15 °C/min → 290 °C → 5 °C/min → 320 °C (2 min)
**MS parameters**	
Interface temperature	280 °C
Ion source temperature	230 °C
Loop time	0.3 s
Measurement mode	Multiple reaction monitoring (MRM)
  2. Detect the 17 sterols by MS/MS under multiple reaction monitoring (MRM) mode. Retention time, collision energies, quantification ions, and confirmation ions for each sterol are shown in Table 2. Figure 2 shows MRM chromatograms for respective sterols.
    **Table 2.** Retention time, quantification ions, confirmation ions, and collision energy (CE) for the sterol analysis

Sterols	Retention time (min)	Quantification ions		Confirmation ions	
		**MRM transition (m/z)**	**CE (eV)**	**MRM transition (m/z)**	**CE (eV)**
7α-hydroxycholesterol	8.48	456 > 208.3	18	456 > 119, 456 > 95.2	30, 33
Cholesterol-d7	8.89	336 > 121.1	18	375 > 145.1, 336 > 109.2	18, 18
Cholesterol	8.94	368 > 145.1	21	329 > 95.1, 329 > 81.1	27, 24
Desmosterol	9.24	129 > 73	15	129 > 57.9, 129 > 127.1	30, 15
7-dehydrocholesterol	9.28	351 > 145.1	30	351 > 128.1	42
7β-hydroxycholesterol	9.38	456 > 233.2	18	233 > 73.1	24
Lathosterol	9.41	213 > 157.2	12	213 > 81.1	15
4β-hydroxycholesterol	9.61	366 > 158.3	18	366 > 129.1	36
24,25-dihydrolanosterol	10.01	395 > 145.2	30	395 > 107	30
7α,25-dihydroxycholesterol	10.21	131 > 73.1	18	544 > 73.2	39
Lanosterol	10.36	393 > 95.2	24	393 > 187.4	12
24,25-epoxycholesterol	10.44	143 > 73.1	18	129 > 73.1, 143 > 128.1	15, 18
24(S)-hydroxycholesterol	10.60	159 > 69.1	9	159 > 73.1	18
7α,27-dihydroxycholesterol	10.60	103 > 73.1	9	544 > 233.2	33
25-hydroxycholesterol-d6	10.75	137 > 73.2	15	137 > 58.2	30
25-hydroxycholesterol	10.79	131 > 73.2	9	131 > 58.1	30
27-hydroxycholesterol	11.26	129 > 73.1	15	456 > 131.2, 417 > 69	42, 42
*Notes:*
  - *Cholesterol-d7 and 25-hydroxycholesterol-d6 serve as internal standards for non-hydroxylated sterols and hydroxylated sterols, respectively.*
  - *The quantification ions are used to identify and calculate the amount of each sterol. The confirmation ions are for assisting in the identification of respective sterols. Several sterols share the same precursor ions, quantification ions, and confirmation ions. When these sterols are not resolved, distinct precursor ions should be selected for the identification and quantification.*
  - *Precursor ions for quantification and confirmation ions are carefully determined by the following criterion: higher m/z and stronger intensity. Collision energy is automatically calculated by the instrument.*
  - *Retention times for each sterol become shorter after a column is cut for maintenance because it depends on the column length.*
- **Cellular protein assay**
  1. After cellular lipid extraction (step B5), add 1 mL of 0.1 N NaOH (see Recipes) into each well and place at room temperature for 20–24 h to solubilize cellular protein.
  2. Mix solubilized proteins using a pipette and transfer 10 μL of a sample to a well of a 96-well plate.
  3. Add 150 μL of BCA assay reagent to each well, incubate the plate for 30 min at 37 °C, and record absorbance at 562 nm using a plate reader (SpectraMax M2e).
  4. Determine the protein concentration of samples with bovine serum albumin as a standard.
  **Figure 2.**
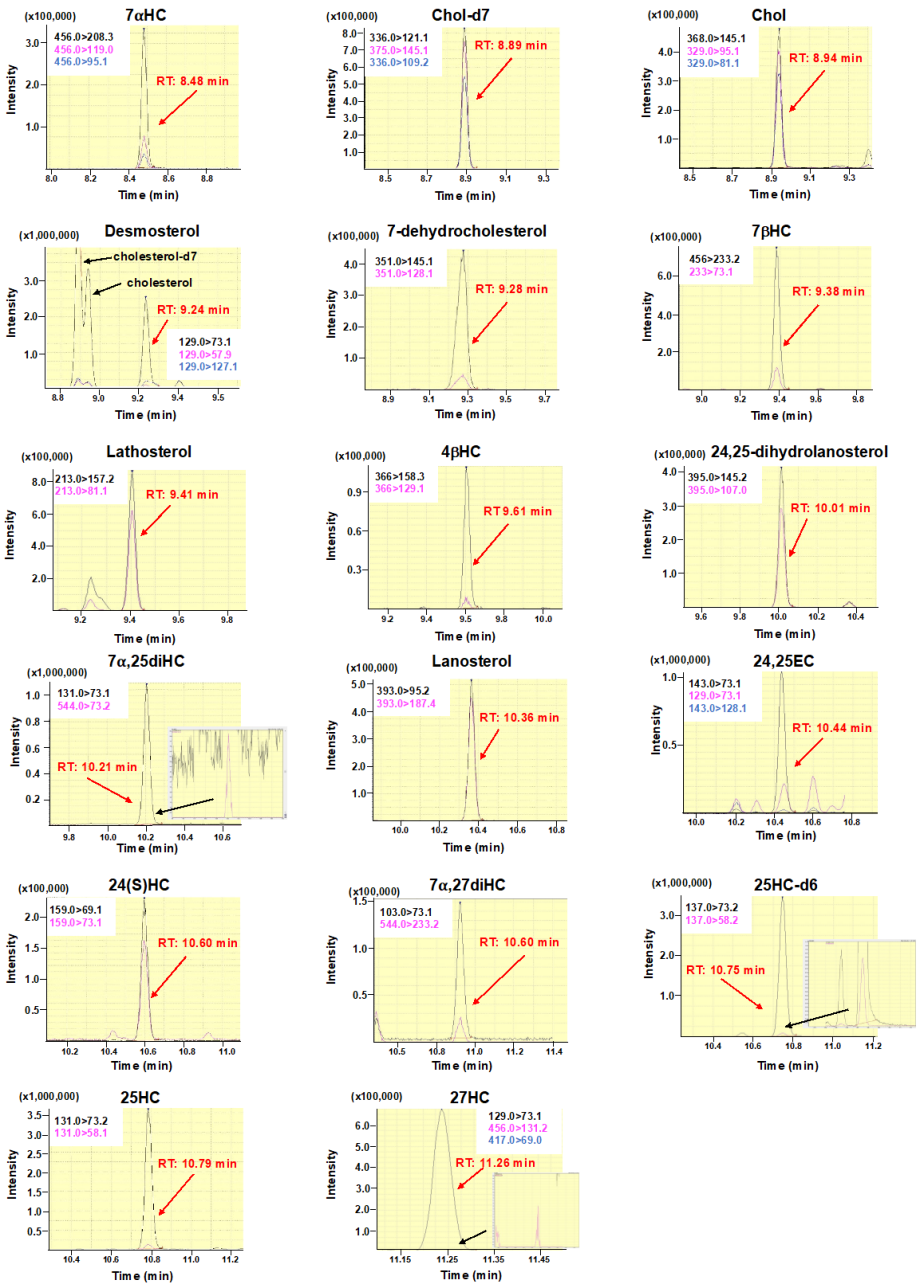
Multiple reaction monitoring (MRM) chromatograms of sterols analyzed in this protocol. MRM chromatograms for each sterol are shown according to retention time. Quantification ions (black) and confirmation ions (pink and blue) are presented in each chromatogram. Black lines and pink and blue lines denote quantification ions and confirmation ions, respectively.

## Data analysis

**Figure 3.**
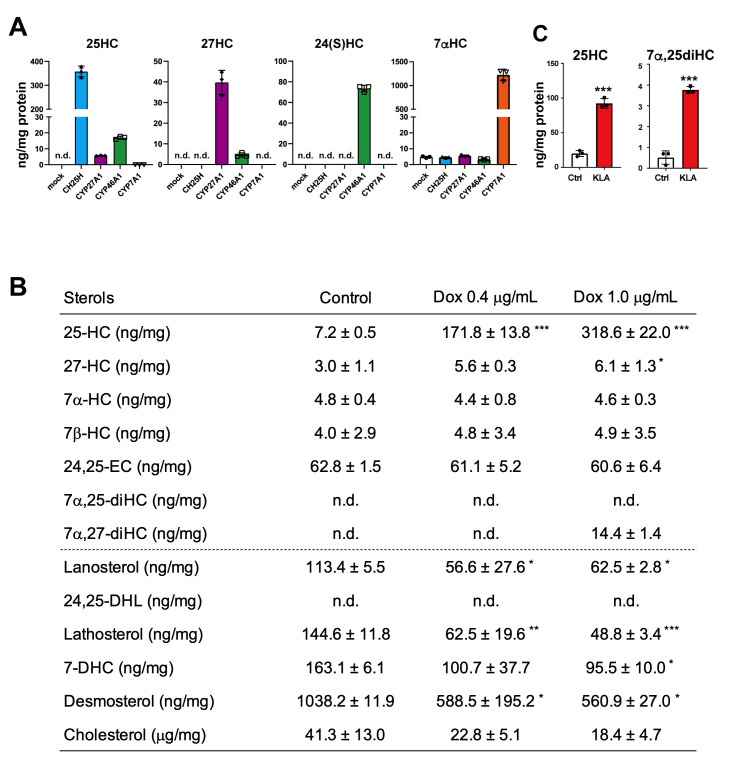
Comprehensive and quantitative determination by GC–MS/MS of oxysterols synthesized by cholesterol hydroxylases in living cells. (A) Oxysterol contents in CHO-K1 cells forcedly expressing CH25H, CYP27A1, CYP46A1, or CYP7A1. The data show that CH25H and CYP7A1 are very specific to synthesizing 25-HC and 7α-HC, respectively, while CYP27A1 and CYP46A1 exhibit broader specificities; CYP27A1 produces not only 27-HC but also 25-HC, and CYP46A1 synthesizes 25-HC and 27-HC in addition to 24(S)-HC. (B) CH25H expression level–dependent production of 25-HC and its effect on intermediate sterol contents in CHO-CH25H^ tet-on^ cells. Doxycycline (Dox) induces 25-HC synthesis in a dose-dependent manner, which results in the reduction in intermediate sterol contents. 24,25-DHL, 24,25-dihydrolanosterol; 7-DHC, 7-dehydrocholesterol. (C) 25-HC and 7α,25-diHC contents in J774.1 murine macrophages treated with or without 100 ng/mL of Kdo2-Lipid A (KLA) for 20 h. Stimulation of the cells with KLA induces the expression of CH25H and the production of 25-HC and 7α,25-diHC. Statistical analyses were performed by one-way ANOVA with Dunnett post-hoc test (B) or Student’s *t*-test (C) (*p < 0.05, **p < 0.01, ***p < 0.001). Data presented in this figure were reproduced from the original paper (Saito et al., 2023).

1. Obtain each sterol peak area using GC-MSsolution v4 software.
2. Create a standard curve for each sterol using GC-MSsolution. Typical standard curves for individual sterols are shown in Supplementary Figure 1.
3. Determine the concentration of each sterol in a sample using GC-MSsolution based on peak areas and the standard curve.
*Note: As described in Procedure D, the ranges of the two standard curves are as follows: 1 ng/mL to 1 μg/mL for non-hydroxysterols and 0.1 ng/mL to 200 ng/mL for oxysterols.*
4. Find the extraction efficiency of cholesterol-d7 and 25-hydroxycholesterol-d6 with the following equation:
$$E=\frac{C\;\times \;V\;\times \;1000}{I\;}$$
  where *E* is extraction efficiency, *C* is the concentration (μg/mL) of cholesterol-d7 or 25-hydroxycholesterol-d6 detected by GC–MS/MS, V is the final volume (mL) of the sample (0.1 mL in this protocol), and *I* is the quantity (ng) of the internal standard added at the step B6 (100 ng of cholesterol-d7 or 10 ng of 25-hydroxycholeterol-d6, in this protocol).
5. Calculate the original amounts of each sterol per sample using the appropriate extraction efficiency.
*Note: The extraction efficiencies of cholesterol-d7 and 25-hydroxycholesterol-d6 are used to calculate the original amounts of non-hydroxysterols and oxysterols, respectively.*
6. Normalize the amounts of each sterol to cell protein per well (Figure 3).
7. All data are represented by the mean ± S.D. of at least three independent biological replicates. Statistical analyses are performed with RStudio software by Student’s *t*-test or one-way ANOVA with Dunnett or Tukey-Kramer post-hoc test. *p* < 0.05 is considered statistically significant.

**Advantages and limitations**

LC–MS/MS, GC–MS, and GC–MS/MS have been employed for sterol analysis, with GC–MS as the most traditional technique (Krone et al., 2010; McDonald et al., 2012; Griffiths et al., 2013; Saito et al., 2023). Although each method has advantages and limitations, the current GC–MS/MS protocol described above has several advantages over other methods. Since our method is highly sensitive, as 1 pg of sterols can be detected, lipids extracted from cells in a well of a 6-well plate (approximately 0.5–1 × 10^ 6^ cells) are sufficient to measure a series of sterols, including oxysterols and intermediate sterols. The current GC–MS/MS analysis of cellular sterol is much more sensitive than our previous analysis with GC–MS, in which cellular lipids were extracted from a 100 mm dish or a whole 6-well plate (Yamauchi et al., 2007), while the amounts of cellular sterol contents detected are equivalent. Therefore, many samples can simultaneously be handled for studying multiple experimental conditions in a single assay. In addition, the run time for a sample in our method is approximately 15 min. Typical run times for sterol analysis by GC–MS and LC–MS/MS are 20–30 min and 12–20 min, respectively, depending on the columns and conditions used. Thus, our GC–MS/MS method is comparable to LC–MS/MS analysis concerning run time.

Furthermore, GC-based methods generally show better chromatographic resolution than LC–MS/MS. The previous LC–MS/MS method was unable to resolve 7α-HC and 7β-HC (McDonald et al., 2012), whereas our GC–MS/MS protocol distinguished between these two oxysterols (Saito et al., 2023). As such, GC–MS/MS is the superior technology for analyzing small amounts of isomeric oxysterols, while a major limitation of LC–MS/MS methods is that such oxysterols tend to provide similar spectra.

However, GC–MS/MS-based sterol analysis also has disadvantages. In contrast to LC–MS/MS methods, which do not often require derivatization (McDonald et al., 2012), sterol needs to be derivatized for GC–MS/MS analysis, which is a laborious and time-consuming process. In addition, since GC–MS/MS detects a compound of interest with MRM mode like LC–MS/MS, only target molecules with available standards can be measured.

In summary, the current GC–MS/MS protocol provides a rapid and highly sensitive method for quantification of a series of oxysterols synthesized endogenously within cells, determining cholesterol hydroxylase activity quantitatively.

## Supplementary information

The following supporting information can be downloaded here:
